# Accidental Entrapment of Electrical Mapping Catheter by Chiari's Network in Right Atrium during Catheter Ablation Procedure

**DOI:** 10.1155/2016/1302473

**Published:** 2016-06-05

**Authors:** Atsushi Sakamoto, Tsuyoshi Urushida, Tomoaki Sakakibara, Makoto Sano, Kenichiro Suwa, Takeji Saitoh, Masao Saotome, Hideki Katoh, Hiroshi Satoh, Hideharu Hayashi

**Affiliations:** Division of Cardiology, Internal Medicine III, Hamamatsu University School of Medicine, 1-20-1 Handayama, Higashi-ku, Hamamatsu 431-3192, Japan

## Abstract

A 78-year-old male was admitted to our hospital due to frequent palpitation. His electrocardiogram (ECG) presented regular narrow QRS tachycardia with 170 bpm, and catheter ablation was planned. During electroanatomical mapping of the right atrium (RA) with a multiloop mapping catheter, the catheter head was entrapped nearby the ostium of inferior vena cava. Rotation and traction of the catheter failed to detach the catheter head from the RA wall. Exfoliation of connective tissue twined around catheter tip by forceps, which were designed for endomyocardial biopsy, succeeded to retract and remove the catheter. Postprocedural echocardiography and pathologic examination proved the existence of Chiari's network. The handling of complex catheters in the RA has a potential risk of entrapment with Chiari's network.

## 1. Introduction

Congenital cardiovascular abnormalities have a potential for causing several complications in routine catheter procedure. Chiari's network, the fetal remnant of venous valve in the right atrium (RA), is one of the malformations [1]. We report a case of accidental entrapment of electrode catheter by Chiari's network during catheter ablation procedure.

## 2. Case Report

**Figure 1:**
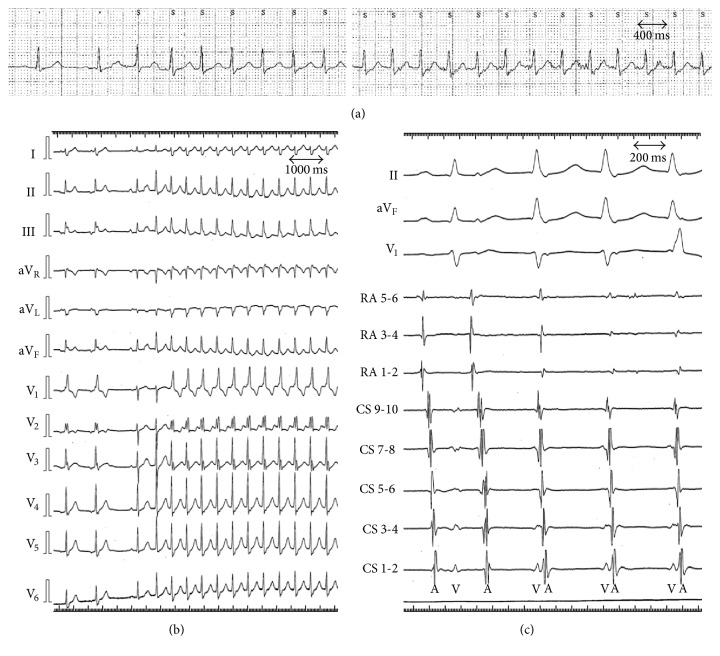
(a) Regular 170 bpm narrow QRS tachycardia detected by Holter electrocardiography (ECG). (b) 12-lead ECG of the supraventricular tachycardia (SVT) (133 bpm) induced by an attachment of electrode catheter tip to the RA wall. 12-lead ECG showed intermittent complete right bundle branch block during both sinus rhythm and SVT. (c) Intracardiac ECG of SVT recorded during the catheter entrapment. SVT was induced with jump up phenomenon. We did not record His bundle electrocardiogram (HBE) to avoid further catheter entrapment. A: atrial electrogram, CS: coronary sinus, RA: right atrium, and V: ventricular electrogram.

A 78-year-old male was referred to our hospital because of frequent palpitation. Holter electrocardiography (ECG) revealed a narrow QRS tachycardia with regular 170 beats per minute (bpm) (Figure 1(a)). His 12-lead ECG showed normal sinus rhythm and intermittent complete right bundle branch block. The transthoracic echocardiography (TTE) showed normal ventricular function and no remarkable abnormality in intracardiac structure, although the observation was quite limited by attenuation artifacts caused by ribs and lung.

After written informed consent was obtained, he underwent an electrophysiological study (EPS) under weak sedation with intravenous infusion of midazolam. Initially, a multipolar electrode catheter was introduced from the right femoral vein and positioned at the coronary sinus (CS). When the tip of electrode catheter attached to the RA wall, a supraventricular tachycardia (SVT) with heart rate of 133 bpm was induced immediately after the jump-up phenomenon (Figures 1(b) and 1(c)), suggesting a slow-fast atrioventricular nodal reentrant tachycardia. However, since the heart rate of previously detected SVT was 170 bpm, which was quite higher than the catheter-induced SVT, this patient had potentially two or more different types of SVT. Furthermore, this patient had a small Koch's triangle, and the precise anatomical location of His bundle during slow pathway potential ablation had to be confirmed to avoid procedure induced atrioventricular block. Thus, we decided to use electroanatomical 3D mapping system (EnSite Velocity system, St. Jude Medical, St. Paul, Minnesota). To make geometry of the RA, we used Reflexion HD*™* catheter (St. Jude Medical, St. Paul, Minnesota), a duodecapolar multiloop intracardiac mapping catheter, through Agilis*™* steerable guiding introducer (St. Jude Medical, St. Paul, Minnesota).

During an electroanatomical mapping of the RA, the head of mapping catheter was entrapped nearby the ostium of inferior vena cava. Rotation and traction of the catheter failed to detach the catheter head from the RA wall. Because frequent SVTs which were provoked by mechanically induced supraventricular premature beats disturbed our catheter removal procedure, we initially tried to treat SVT.

Since the intracardiac ECG at the initiation and during SVT was compatible with slow-fast atrioventricular nodal reentrant tachycardia (Figure 1(c)), the radiofrequency (RF) application targeting the inferior side of Koch's triangle, the anatomical approach of the slow pathway, was delivered targeting at 50 degrees with a maximum setting of 30 W (Blazer II, Boston Scientific, Natick, Massachusetts) with careful attention not to induce atrioventricular block. After 4 times of RF application, the SVT could never be induced.

**Figure 2:**
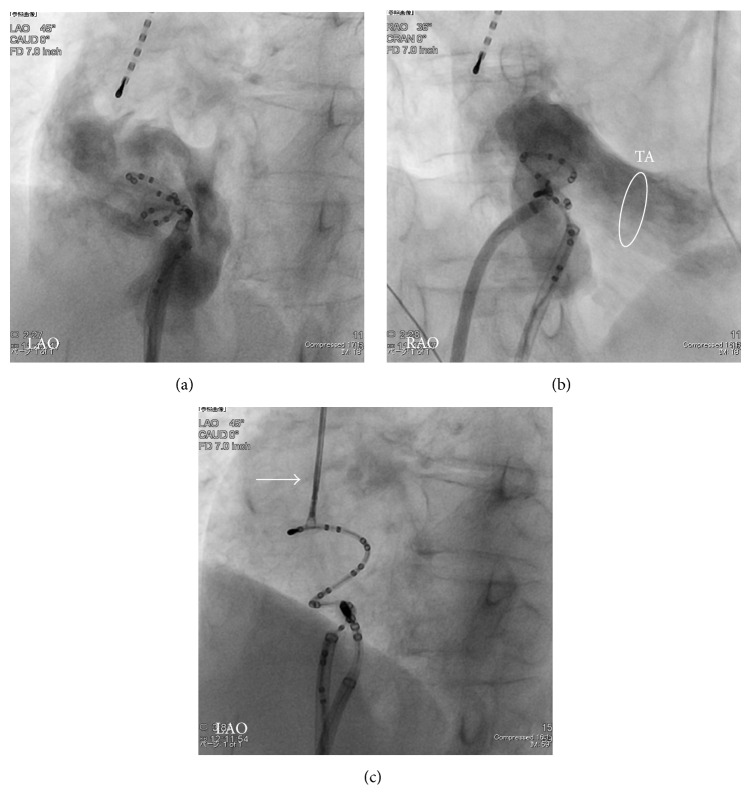
The images of fluoroscopy. Right atriography revealed the entrapped catheter was distant from tricuspid valve, posterior wall of right atrium (RA) in left anterior oblique (LAO) view (a), and lateral wall of RA in right anterior oblique (RAO) view (b). The head of multiloop mapping catheter was apart from tricuspid annulus (TA). The forceps (arrow) designed for endomyocardial biopsy caught the head of multiloop mapping catheter and twined connective tissues (c).

**Figure 3:**
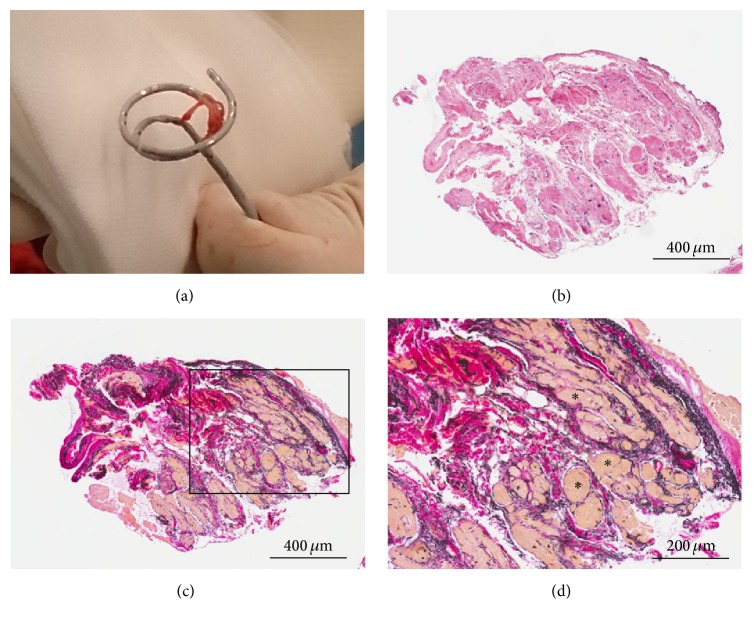
(a) The macroscopic image of removed multiloop mapping catheter. Fascicular connective tissue twined around the catheter tip. (b, c, d) The microscopic images of the removed tissue; (b) low-power field with Hematoxylin Eosin staining, (c) low-power field with Elastica van Gieson staining, and (d) high-power field of Elastica van Gieson staining. They consist of interstitial tissue including elastic fibers and patchy distribution of cardiomyocytes (*∗*).

Right atriography revealed that the entrapped catheter was distant from tricuspid valve and the catheter traction did not deform the RA shadow (Figures 2(a) and 2(b)). The traction of the entrapped catheter did not induce supraventricular or ventricular premature beats. Thus, we decided to pull apart the catheter tip from twined connective tissues by using the forceps which were designed for endomyocardial biopsy (6 Fr 105 cm Biopsy Forceps, Technowood, Tokyo). We initially exfoliated the connective tissue twined around the catheter tip using the forceps (Figure 2(c)), pulled the catheter vigorously, and finally succeeded to retract and remove the catheter without any complications and surgical intervention. In the macroscopic finding, the fascicular connective tissue twined around the tip of the multiloop mapping catheter (Figure 3(a)). Later, the microscopic examination revealed an accumulation of interstitial tissue including elastic fiber and a patchy distribution of cardiomyocytes (Figures 3(b)–3(d)), which are compatible with Chiari's network [2].

**Figure 4:**
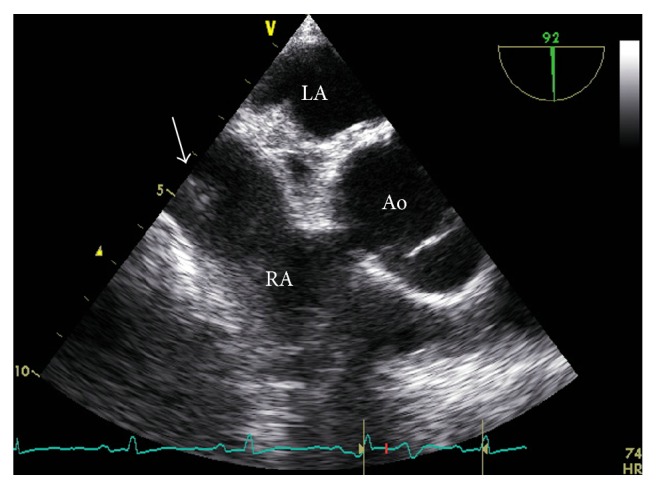
The postprocedural transesophageal echocardiogram (TEE) findings showed the residual floating structure in the RA (arrow), suggesting the tissue of Chiari's network. Ao: ascending aorta, LA: left atrium, and RA: right atrium.

Finally, we confirmed that the dual atrioventricular nodal physiology disappeared and any SVTs were not induced even under the isoproterenol infusion. The postprocedural transesophageal echocardiography (TEE) showed residual floating structures in the RA (Figure 4), suggesting the disrupted tissue of Chiari's network.

## 3. Discussion

Chiari's network is a congenital anatomical variation at the junction of the RA and superior and inferior vena cava. In 1897, Hans Chiari described the intricate fenestrated reticulum containing multiple threads inserting on the anterior surface of Eustachian valve, the posterior wall of the ostial inferior vena cava, crista terminalis, and the tubercle of Lower [1]. A fenestrated valve of CS (Thebesian valve) has also been called Chiari's net. This structure is considered as embryological remnants of right valve of the sinus venosus and septum spurium that persisted after embryological development [1].

The prevalence of Chiari's network is reported to be approximately 2% with estimates varying from 1.3 to 4% in postmortem findings and 0.3 to 9.5% in TTE findings [1]. The previously reported clinical complications associated with Chiari's network are the intra-RA thrombus formation [3] as well as the entrapment of thromboemboli [4], atrial tachyarrhythmia [5], and device entrapment [6–12]. To date, 9 cases of device entrapment with Chiari's network have been reported. Entrapped devices were guidewires [6–8], pacemaker leads [9], right heart diagnostic catheter [11], atrial septal defect (ASD) closure device [10], and electrical mapping catheter [12]. Surgical resections were required in two cases [11, 12].

The Reflexion HD*™* catheter is a multiloop intracardiac mapping catheter that is designed to create highly detailed geometries and electrical maps in 3D mapping system. Although, at present, there is no report that this catheter is entrapped with intracardiac structure, it is likely that the catheter could twine toward fascicular structures due to the multispiral designs. In this case, the mobility of the catheter head in the RA was restricted from the beginning of intra-RA mapping. The catheter tip possibly twisted around the Chiari's network just when the catheter was inserted to the RA. A number of rotations of the catheter for intra-RA mapping might make more complex binding to fascicular tissue and the catheter.

This is a rare report of a successful bail out of catheter entrapment in Chiari's network using the forceps. We recognized the risk of further complication such as the RA wall perforation and disruption of tricuspid valve by using the forceps in the RA [13, 14]. The traction of the entrapped catheter did not induce supraventricular and ventricular premature beats or deform the RA wall. Furthermore, the entrapped catheter was distant from tricuspid valve in the right atriography (Figure 2(b)). We considered that the risk of further complications was low and fortunately we succeeded to remove the catheter without any surgical intervention.

In conclusion, the handling of complex catheters in the RA has a potential risk of entrapment with Chiari's network. A careful observation of the RA prior to catheter ablation is recommended to prevent this complication.

## References

[1] Loukas M., Sullivan A., Tubbs R. S., Weinhaus A. J., DerDerian T., Hanna M. (2010). Chiari's network: review of the literature. *Surgical and Radiologic Anatomy*.

[2] Araki T., Takasugi M., Otsuki S. (2012). Case report of resection of Chiari network for severe tricuspid regurgitation. *Shinzo*.

[3] Benbow E. W., Love E. M., Love H. G., MacCallum P. K. (1987). Massive right atrial thrombus associated with a Chiari network and a Hickman catheter. *American Journal of Clinical Pathology*.

[4] Obaji S. G., Cooper R., Somauroo J. (2012). Chiari network: a protective filter against pulmonary embolism in a case of polycythaemia. *BMJ Case Reports*.

[5] Prajapat L., Ariyarajah V., Spodick D. H. (2007). Abnormal atrial depolarization associated with chiari network?. *Cardiology*.

[6] Shimoike E., Ueda N., Maruyama T., Kaji Y., Niho Y. (2001). Entrapment of a guide wire by the Chiari network in a patient with ablated idiopathic ventricular tachycardia. *Journal of Interventional Cardiac Electrophysiology*.

[7] Aydin A., Gürol T., Yilmazer M. S. (2011). Catheter entrapment around the chiari network during percutaneous atrial septal defect closure. *The Anatolian Journal of Cardiology*.

[8] Hightower J. S., Taylor A. G., Ursell P. C., Laberge J. M. (2015). The chiari network: a rare cause of intracardiac guide wire entrapment. *Journal of Vascular and Interventional Radiology*.

[9] Maruyama T., Kurogouchi F. (2004). Entrapment of a tined lead by the chiari network with preserved atrial sensing ability in a patient with atrioventricular block: a case report. *Journal of Cardiology*.

[10] Cooke J. C., Gelman J. S., Harper R. W. (1999). Chiari network entanglement and herniation into the left atrium by an atrial septal defect occluder device. *Journal of the American Society of Echocardiography*.

[11] Goldschlager A., Goldschlager N., Brewster H., Kaplan J. (1972). Catheter entrapment in a Chiari network involving an atrial septal defect. *Chest*.

[12] Grecu M., Floria M., Tinică G. (2013). Complication due to entrapment in the Chiari apparatus. *Europace*.

[13] Chimenti C., Frustaci A. (2013). Contribution and risks of left ventricular endomyocardial biopsy in patients with cardiomyopathies: a retrospective study over a 28-year period. *Circulation*.

[14] Fiorelli A. I., Coelho G. H., Aiello V. D. (2012). Tricuspid valve injury after heart transplantation due to endomyocardial biopsy: an analysis of 3550 biopsies. *Transplantation Proceedings*.

